# A novel commensal *Neisseria* species harboring the gonococcal diagnostic marker DR-9 causes false-positive Roche cobas NAAT results

**DOI:** 10.1128/jcm.01022-25

**Published:** 2025-11-19

**Authors:** Julio C. Ayala, Shelby M. Hutton, John C. Cartee, Jennifer L. Reimche, Sandeep J. Joseph, Bridgett Herrod, Halie LaPoint, Linda Cao, Tamara Baldwin, Youli Gainey, Aaron C. Ermel, James A. Williams, Elizabeth Palavecino, Sancta B. St Cyr, Matthew W. Schmerer, Ellen N. Kersh

**Affiliations:** 1 Division of STD Prevention, Centers for Disease Control and Prevention 1242, Atlanta, Georgia, USA; 2 Clinical Bacteriology and STD Teams, Texas Department of State Health Services 8193, Austin, Texas, USA; 3 Department of Internal Medicine, Division of Infectious Diseases, Indiana University School of Medicine 12250, Indianapolis, Indiana, USA; 4 Department of Pathology, Atrium Health Wake Forest Baptist Medical Center 12280 , Winston-Salem, North Carolina, USA; Marquette University, Milwaukee, Wisconsin, USA

**Keywords:** commensal *Neisseria* species, *Neisseria gonorrhoeae*, Ng NAAT, gonorrhea diagnosis, DR-9

## Abstract

*Neisseria gonorrhoeae* (*Ng*), the etiologic agent of gonorrhea, is the second most reported bacterial sexually transmitted infection globally. In the USA, nucleic acid amplification tests (NAATs) are considered the gold standard for diagnosis. Although NAATs are sensitive and offer high throughput, cross-reactivity with commensal *Neisseria* can compromise specificity. Here, we report the isolation and characterization of a novel commensal *Neisseria* sp. from the oropharynx of a patient with suspected gonococcal treatment failure. An initial diagnosis was made using the Roche cobas CT/NG test on the cobas 8800 system, which repeatedly yielded positive results post-treatment. A confirmatory Aptima Combo 2 (AC2, Hologic) test was negative, and multiple culture attempts failed to isolate *Ng*. Instead, a commensal *Neisseria* strain was recovered. Whole-genome sequencing revealed this isolate shared partial genomic identity with several *Neisseria* spp., including *Ng*, and carried a distinct region encoding the gonococcal diagnostic marker DR-9, the target of cobas assays. Cross-reactivity was confirmed with the cobas 4800 and 6800 platforms, but not with the AC2 assay targeting 16S rRNA. We discuss the possible origins of the DR-9 marker on this strain as well as examine its antimicrobial susceptibility profile and genomic resistance markers. These findings highlight the potential for misdiagnosis and unnecessary treatment when commensal *Neisseria* species harbor *Ng* diagnostic targets. Our study underscores the need for genomic surveillance of *Ng* and commensal *Neisseria*, not only to monitor diagnostic performance but also to track commensals that may serve as reservoirs of antimicrobial resistance determinants and contribute to the spread of resistance.

**IMPORTANCE:**

Accurate diagnosis of gonorrhea is critical for effective treatment and antimicrobial stewardship. Nucleic acid amplification tests, the mainstay of gonococcal diagnostic testing, can yield false-positive results due to genetic overlap between *Neisseria gonorrhoeae* and commensal *Neisseria* species, especially from extragenital sites like the oropharynx. Prior studies, such as Hopkins et al. (2023), have recognized this limitation and proposed supplemental tests to improve specificity for oropharyngeal specimens. Here, we describe a novel commensal *Neisseria* strain isolated from a patient with suspected treatment failure that harbors the gonococcal diagnostic marker DR-9. This case highlights the need for confirmatory testing using an alternate gene target in cases where repeated positive tests are obtained with extragenital specimens and demonstrates the need for improved test specificity, particularly for anatomical sites such as the pharynx, which has high commensal diversity. Enhanced molecular surveillance of commensal *Neisseria* populations will be vital for understanding and minimizing diagnostic cross-reactivity.

## INTRODUCTION

*Neisseria gonorrhoeae* (*Ng*), the etiologic agent of gonorrhea, remains a significant public health concern due to its high prevalence and increasing resistance to antibiotics (1–3). Nucleic acid amplification tests (NAATs) have become the standard for the diagnosis of *Ng* infections due to their high sensitivity and specificity (4, 5). However, the specificity of these assays can be compromised by the genetic similarity between *Ng* and commensal *Neisseria* species, particularly those residing in the oropharynx (6). This genetic overlap can lead to false-positive results, complicating diagnosis and treatment strategies (7–9).

The direct-repeat region 9 (DR-9) targeted by the Roche cobas NAATs is one such genetic element that can be shared between *Ng* and commensal *Neisseria* species (10, 11). The presence of DR-9 in commensal species raises concerns about the potential for cross-reactivity in these diagnostic assays. Moreover, commensal *Neisseria* species have been identified as reservoirs of antimicrobial resistance (AMR) genes, which can be transferred to pathogenic *Neisseria* through horizontal gene transfer (HGT) mechanisms. This transfer not only complicates treatment options but also poses a threat to public health by facilitating the spread of resistance (6, 12, 13).

In this study, we report the identification of a commensal *Neisseria* strain carrying a chromosomal copy of the DR-9 gonococcal marker and causing a false-positive diagnostic result for gonorrhea, representing only the second such case reported to date. We explore the implications of this finding for diagnostic accuracy, the potential mechanisms of DR-9 acquisition, and the broader context of AMR gene reservoirs in commensal *Neisseria* species.

## MATERIALS AND METHODS

### Strains and media

*N. gonorrhoeae* F18 (ATCC 49226) and *Neisseria* sp. SLRRB23 strains used in this study were grown overnight at 36°C under 5% (vol/vol) CO_2_ on GC agar base plates (BD Difco) containing 1% IsoVitaleX supplement (Becton Dickinson). When required, bacterial cultures were resuspended in GC broth (14).

### NAAT cross-reactivity testing

To prepare bacterial inocula for NAAT cross-reactivity testing, we first precalculated the CFU/mL concentrations by resuspending bacterial biomass from overnight GC agar plate cultures in GC broth to an optical density of 1.0 at 600 nm (OD_600_, determined using a UV-Visible Spectrophotometer Spectronic 200 E). Then, we plated 100 µL of 10-fold serial dilutions of the resuspended bacterial solution on GC agar and performed colony counting after 16–24 h of incubation. Based on this precalculation, bacteria from GC agar plates were then resuspended in GC broth and diluted to clinically relevant concentrations into cobas PCR Media Uni Swab Sample tubes (Roche Molecular Systems, Inc.) for the cobas CT/NG v.2.0 test on the 4800 system and the cobas CT/NG test on the 6800 system; for side-by-side comparison on a NAAT platform targeting a different gene, we also added precalculated concentrations of the bacteria into Aptima Urine Specimen Collection Tubes (Hologic) for the Aptima Combo 2 (AC2) test on the Hologic Panther Fusion System (Table 1). Samples were tested on the cobas 4800 test system at the Infectious Diseases Laboratory, Indiana University School of Medicine, Indiana, on the cobas 6800 test system at the Department of Pathology, Atrium Health Wake Forest Baptist Medical Center, North Carolina, and on a Hologic Panther Fusion test system at the Centers for Disease Control and Prevention (CDC)’s STD Reference and Research Laboratory (SLRRB) in Atlanta, GA.

**TABLE 1:** Cross-reactivity of the commensal isolate *Neisseria* sp. SLRRB23 with three FDA-approved gonorrhea diagnostic NAATs^*a*^^,^^*b*^

Colony-forming units/mL	Concentration simulating infection type	Sample	Cobas CT/NG on the 4800 test platform			Cobas CT/NG on the 6800 test platform			Aptima Combo 2 (CT/NG) on the Hologic Panther	
			NG Ct value^*c*^	IC Ct value	NG qualitative result	NG Ct value^*c*^	IC Ct value	NG qualitative result	Sample RLU (×1,000)^*c*^	NG qualitative result
1.6 × 10^3^	Oropharyngeal	*Neisseria* sp. SLRRB23	32.50	33.20	Positive	30.57	28.78	Positive	13	Negative
4.6 × 10^6^	Symptomatic male urethra	*Neisseria* sp. SLRRB23	21.20	34.10	Positive	18.74	28.71	Positive	15	Negative
3.1 × 10^4^	Male urine	*Neisseria* sp. SLRRB23	27.80	32.70	Positive	24.89	28.67	Positive	9	Negative
10^8^	Arbitrary high concentration	*Neisseria* sp. SLRRB23	18.80	34.20	Positive	14.98	29.24	Positive	22	Negative
1.6 × 10^3^	Oropharyngeal	*Ng* F18	28.80	33.10	Positive	29	28.66	Positive	1,237	Positive
4.6 × 10^6^	Symptomatic male urethra	*Ng* F18	17.00	34.50	Positive	15.31	28.80	Positive	1,221	Positive
3.1 × 10^4^	Male urine	*Ng* F18	24.70	33.30	Positive	22.51	28.78	Positive	1,227	Positive
10^8^	Arbitrary high concentration	*Ng* F18	14.00	34.40	Positive	11.55	29.77	Positive	1,210	Positive
0	n/a	GC broth	n/a	34.20	Negative	n/a	28.65	Negative	11	Negative

^*a*^ Roche cobas Uni Swab and Aptima Urine Specimen Collection Tubes were spiked with *Neisseria* sp. SLRRB23 at different concentrations equivalent to the average gonococcal loads in different human clinical specimens (15, 16). Tests were conducted according to the manufacturer’s instructions and local CLIA regulations. *Ng* F18 and sterile GC broth were included as positive and negative controls.

^*b*^ IC, internal control; Ct, cycle threshold: NG, *Neisseria gonorrhoeae*; RLU = relative light units.

^*c*^ Assay Ct cutoff values (for *Ng*) are defined as 45 for the cobas CT/NG v.2.0 test (4800 platform) and 50 for the cobas CT/NG test for use on the cobas 6800/8800 Systems. For the Aptima Combo 2 assay, the RLU positivity range for GC testing only is between 150 and <4,500.

### Antimicrobial susceptibility testing (AST)

AST was performed using Etest antibiotic strips (Biomerieux, France) on GC agar base plates supplemented with 1% IsoVitaleX, as previously described (17). Minimum inhibitory concentrations (MICs) were read after 20–24 h incubation at 36°C under 5% (vol/vol) CO_2_.

### Whole-genome sequencing (WGS) and bioinformatic analysis

WGS was performed at the CDC, as previously described (18). Briefly, genomic DNA was extracted from overnight GC agar-grown bacteria using the Wizard Genomic DNA Purification Kit (Promega, Madison, WI). Libraries for WGS were prepared using the Nextera XT library preparation kit (Illumina, San Diego, CA), following the manufacturer’s recommendations. Prepared DNA libraries were sequenced on the MiSeq (Illumina) platform using V2 reagents (2 × 250 bp). The quality of the Illumina reads was assessed using FastQC. CutAdapt v.1.8.3 was used to trim reads and to remove adapter sequences and bases using a minimum quality cutoff of <Q30 and a minimum length cutoff of 19. SPAdes v.3.9.0 (19) was used to generate a *de novo* genome assembly from the sequencing reads under default parameters. Prokka (20) was used to generate a gene annotation file (GFF3) using the SPAdes-generated genome FASTA file. Genomic analysis of the antimicrobial resistance genetic profile was performed using an in-house customized Python script called the *Neisseria gonorrhoeae* Genome Profiler and Typing Tool v.2.9.2 (CDC [21]). Paired-end Illumina reads were also mapped to the *Neisseria gonorrhoeae* FA19 reference genome using BWA (22), and coverage statistics were calculated with SAMtools (23), reporting the percentage of the genome with ≥10× depth. Average nucleotide identity (ANI) was estimated using two complementary approaches: FastANI (k-mer-based) (24) and PyANI (BLAST-based) (25). Representative reference genomes from each *Neisseria* species were downloaded from the NCBI RefSeq database, guided by species clustering in the most recent *Neisseria* whole-genome phylogeny (26). Both FastANI and PyANI analyses were performed to generate pairwise ANI estimates between the study isolate, SLRRB23, and other *Neisseria* species. Based on these results, a subset of pathogenic and commensal *Neisseria* species was selected for pan-genome analysis using Roary (27). The resulting core genome alignment was used to infer phylogenetic relationships with RAxML (28), employing the autoMRE bootstrapping criterion and the Gamma-GTR model.

### Molecular analysis of remnant NAAT specimen

In an attempt to identify gonococcal DNA present within a remnant pharyngeal NAAT specimen from the patient (initially tested positive with the cobas CT/NG test on the cobas 8800 platform and submitted in a cobas PCR Media Uni Swab Sample tube [Roche Molecular Systems, Inc.]), we extracted DNA using the QIAamp DNA Mini Kit (QIAGEN), using 400 µL of specimen material and eluting in 100 µL of elution buffer. PCR was carried out using the TaKaRa Ex Taq DNA Polymerase kit (Takara Bio Inc.) on a SimpliAmp thermocycler (Applied Biosystems). We attempted to amplify the *porA* pseudogene, *gyrA, penA,* and 23S rRNA genes using primers designed to amplify conserved regions within *Ng* and to exclude commensal *Neisseria* species (Table S1). PCR products were visualized on ScreenTapes D5000 and D1000 using the TapeStation 2100 Bioanalyzer (Agilent).

## RESULTS

### A case of suspected treatment-resistant oral gonorrhea

In March 2023, a 34-year-old male patient visited a clinic in Austin, Texas, for routine HIV pre-exposure prophylaxis management, which included screening for sexually transmitted infections at all anatomic sites of exposure. He acknowledged condomless oral and anal sex with multiple male partners 2 months prior to his clinic visit and denied ever having any symptoms. Gonorrhea screening was performed using the Roche cobas CT/NG NAAT on the cobas 8800 platform at the Center for Disease Detection (LabCorp Specialty Labs, San Antonio, TX). The results were found to be negative at the urethral and rectal sites, but positive in the oropharynx. The patient was subsequently treated with 500 mg of ceftriaxone intramuscularly according to the current national treatment guidelines for uncomplicated gonorrhea (29). Following initial treatment, the patient continued to test positive on pharyngeal swabs collected at test-of-cure appointments via the cobas assay over the subsequent 3 months. This prompted retreatment, first with ceftriaxone 1 g and later with 240 mg intramuscular gentamicin and 2 g oral azithromycin. Ultimately, an investigation for suspected gonococcal treatment failure was conducted in conjunction with the CDC. Reinfection was determined not to be a cause of the persistently positive results. The patient had one main sexual partner within the jurisdiction and multiple prior casual partners outside of the jurisdiction. His main partner tested negative for gonorrhea and was presumptively treated with ceftriaxone. The patient denied new sexual partners after his initial positive result and remained abstinent throughout the investigation. Prior casual partners were not contacted.

Based on suggested guidance by the CDC, confirmatory testing was performed with the Aptima Combo 2 assay on a new pharyngeal specimen, which returned negative. In June 2023, attempts were made to isolate a gonococcal culture from the patient using ESwab (Copan) and plating onto modified Thayer-Martin agar and using the InTray GC culture detection device (Biomed Diagnostics) at the Texas Department of State Health Services clinical microbiology laboratory in Austin, Texas. Although neither culture method isolated *Ng*, a commensal bacterial strain was recovered from the ESwab and sent, along with a remnant NAAT specimen, to the CDC’s SLRRB for further investigation. Attempts to PCR-amplify genes *penA*, *gyrA*, 23S rRNA, and pseudogene *porA* of the pharyngeal remnant NAAT specimen from the patient failed to confirm the presence of any *N. gonorrhoeae*-specific genetic material (Fig. S1). We were therefore unable to confirm that the patient was infected with *Ng*.

### Species identification of a commensal *Neisseria* isolate reveals a previously uncharacterized species

**Fig 1:**
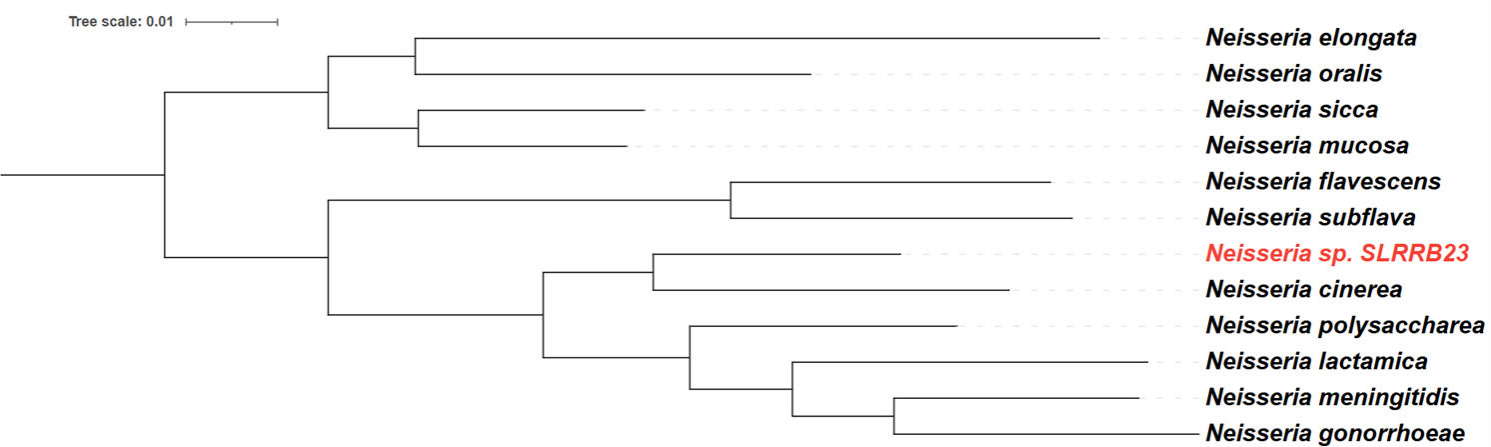
Phylogenetic analysis of human-colonizing *Neisseria* species. Midpoint-rooted maximum-likelihood *Neisseria* species tree based on concatenation of the DNA sequences of 205 core *Neisseria* genes. The novel commensal isolate SLRRB23 is shown in red font, clustered with *N. cinerea*.

The commensal isolate was initially characterized at CDC using matrix-assisted laser desorption ionization-time of flight mass spectrometry (MALDI-TOF MS; Bruker, Germany) and was identified as belonging to the genus *Neisseria,* with inconclusive results at the species level: *N. lactamica* (Bruker MALDI Biotyper identification score 1.87), *N. meningitidis* (1.86), *N. cinerea* (1.85), and *N. subflava* (1.73). These results suggest that the strain may represent a previously uncharacterized *Neisseria* species, as several studies have demonstrated the limitations of MALDI-TOF MS in accurately identifying novel or closely related *Neisseria* strains absent from current reference spectra databases (30, 31). To further confirm the identity of the commensal *Neisseria* isolate, we purified its genomic DNA and performed WGS using the Illumina MiSeq platform. The analysis revealed a bacterial isolate with a genome of approximately 2,125,888 bp (File S1, GenBank accession number: NZ_JBPBFH000000000). We have designated the strain *Neisseria* sp. SLRRB23. We examined the presence of the *ggt* gene, which encodes γ-glutamyl transpeptidase, a biochemical identification marker for *N. meningitidis* (32). This enzyme is phenotypically active only in *N. meningitidis* and exists as a transcriptionally active pseudogene (*ggh*) in *N. gonorrhoeae* (33). BLAST analysis revealed no sequence in the new isolate with similarity to *N. meningitidis ggt*. Furthermore, BLAST searches against the PubMLST database of nonpathogenic *Neisseria* species showed that only 0.34% of all reported commensal *Neisseria* genomes share significant sequence identity with *ggt* (at sequence coverage >25%, identity >75%; Table S2). The absence of *ggt* in this isolate therefore supports the conclusion that it is not *N. meningitidis*. Additionally, paired-end reads mapped poorly to the *Neisseria gonorrhoeae* FA19 reference: only 69.74% of the genome achieved ≥10× depth. This level of coverage is inconsistent with *N. gonorrhoeae* and supports the conclusion that the isolate SLRRB23 is not *Ng*. BLAST nucleotide analysis of the 16S rRNA sequence showed 100% identity to *N. cinerea* strain ATCC 14685, 98.68% to *N. perflava* strain Branham 7078, 98.2% to *N. gonorrhoeae* strain NCTC 8375, and 97.89% to *N. weaveri* strain 8142, among other *Neisseria* species. To further investigate whether the new commensal isolate is related to *N. cinerea* or whether it may constitute an uncharacterized new species, we performed a genome-wide ANI analysis using the fastANI method, which uses k-mer-based mapping (24), and PyANI, which uses sequence alignment-based methods (25). Across a whole-genome data set comprising 28 *Neisseria* species, SLRRB23 showed the highest similarity to *Neisseria cinerea*, with FastANI = 94.12% and PyANI = 93.57% (Table S3). Given that the most widely accepted threshold for species delineation is an ANI value of <95%–96%, below which two genomes are generally considered to represent different species (34, 35), these results suggest SLRRB23 represents either a distinct species or a highly divergent lineage most closely related to *N. cinerea*. From the broader set, 11 human-associated *Neisseria* (pathogens and commensals) were selected for pan-genome analysis with Roary, yielding a core genome of 205 genes. Maximum-likelihood phylogenetic inference from this core alignment placed SLRRB23 in close proximity to *N. cinerea*, mirroring the ANI signal and reinforcing that SLRRB23 is most closely related to *N. cinerea* yet genomically distinct from established species boundaries (Fig. 1). Taken together, results from MALDI-TOF MS identification, genomic, and phylogenetic analyses suggest that the isolate may constitute a novel *Neisseria* species; however, additional taxonomic analyses are necessary to confirm its classification.

### Testing confirms cross-reactivity of commensal *Neisseria* isolate on different Roche cobas platforms

**Fig 2:**
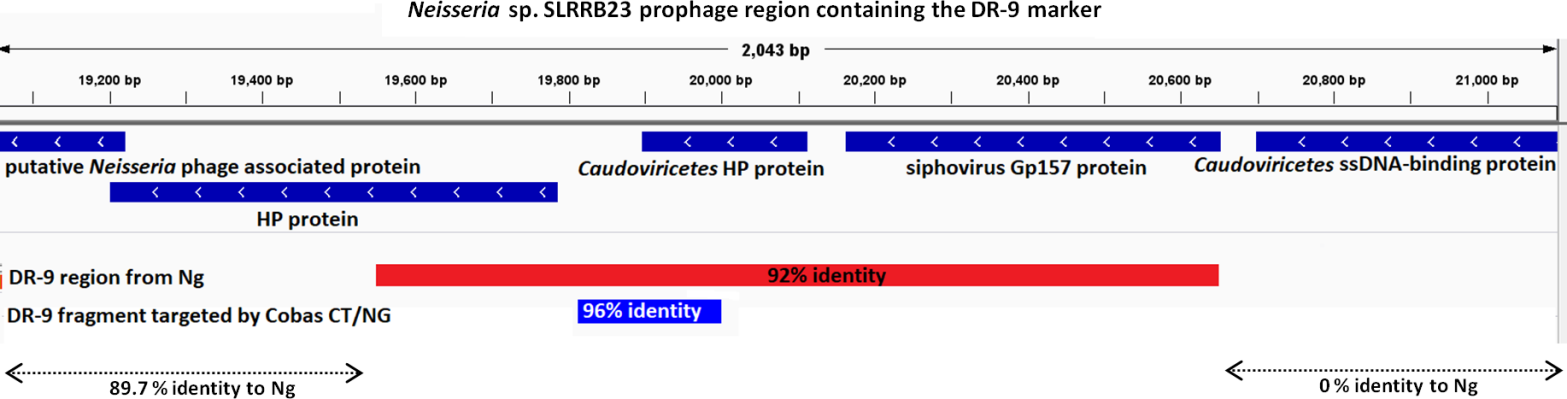
Genomic view of the gonococcal diagnostic target DR-9 within the context of a prophage region of the *Neisseria* sp. SLRRB23 chromosome. Flanking regions of the SLRRB23 DR-9 recombination region (red bar) were tested using BLAST Nucleotide Search to determine the sequence homology to *Ng*, and the results are represented under the double-arrow lines. The genome assembly was done using SPAdes (19) from the Illumina FASTQ files, and the genome annotation file was created using Prokka (20) from the genome assembly FASTA file. The figure was created using Integrative Genomic Viewer (37).

Further genomic analysis showed that strain SLRRB23 contains the DR-9 gonococcal diagnostic target of the Roche cobas CT/NG NAATs (Fig. 2). DR-9 is a highly conserved direct-repeat sequence within the *Ng* genome that is targeted for the diagnosis of *Ng* infections by the FDA-approved Roche cobas NAATs (36). To determine whether the commensal *Neisseria* sp. SLRRB23 cross-reacts with the Roche cobas CT/NG NAATs, we inoculated the isolate in assay-specific swab and urine collection media to concentrations equivalent to the average gonococcal loads present in different human clinical specimens (15, 16) (Table 1). The results confirm that *Neisseria* sp. SLRRB23 cross-reacts with the Roche cobas CT/NG NAAT on both 4800 and 6800 testing platforms, but not with the Aptima Combo 2 NAAT, which targets *Ng* 16S rRNA. Sample tubes containing equivalent concentrations of *N. gonorrhoeae* strain F18 were used as positive controls. The average cycle threshold difference between SLRRB23 and F18 was 3.75 on the cobas 4800 and 2.70 on the cobas 6800, suggesting that the assays exhibit greater affinity for *N. gonorrhoeae* than for the commensal strain, approximately 13-fold and 6-fold higher, respectively.

### The commensal *Neisseria* isolate exhibits non-susceptibility to clinically relevant antibiotics and contains genomic AMR determinants

Given that the patient underwent multiple courses of antibiotic therapy, we hypothesized that the commensal *Neisseria* isolate might exhibit a concerning AMR profile, as previously reported in oropharyngeal *Neisseria* species with the potential to transfer resistance genes to pathogenic *Neisseria* (12, 38). Therefore, we performed AST on strain SLRRB23 using Etests. The results revealed MIC values for azithromycin, cefixime, and ceftriaxone that exceeded the susceptible breakpoints defined by CLSI for *N. gonorrhoeae*, while the tetracycline MIC was markedly above the resistance breakpoint (39) (Table 2).

**TABLE 2:** Antimicrobial susceptibility testing results of *Neisseria* sp. SLRRB23 via Etest^*a*^

Etest	MIC (µg/mL)	Interpretation
Azithromycin	16	Resistant (>1)^*b*^
Cefixime	1	Non-susceptible (>0.25)^*b*^
Ceftriaxone	0.5	Non-susceptible (>0.25)^*b*^
Ciprofloxacin	0.004	Susceptible (≤0.06)^*b*^
Ertapenem	0.125	Undefined
Tetracycline	64	Resistant (≥2)^*b*^
Gentamicin	4	Susceptible (≤4)^*c*^

^*a*^ Etests were conducted according to previously described methods (12), in accordance with manufacturer instructions (Biomerieux, France).

^*b*^ CLSI- or FDA-defined susceptible and resistant breakpoints for *N. gonorrhoeae*.

^*c*^ CDC epidemiological susceptible cutoff value for *N. gonorrhoeae.*

To investigate the genetic basis of this antibiotic resistance profile, we screened the genome of *Neisseria* sp. SLRRB23 for the presence of AMR determinants commonly associated with *N. gonorrhoeae* and other pathogens (Table 3). We identified a mosaic *penA* allele classified as NG-STAR type 59.0001 (with 92.63% sequence identity match), which includes known mutations such as A311V and A501T that are reported to significantly elevate MICs for extended-spectrum cephalosporins (40). In addition, we found a mosaic *mtr* locus carrying a T-to-G transition in the *mtrC* −35 promoter element, generating a more consensus-like −35 sequence (TTGTAT) that enhances expression of the MtrCDE efflux pump (41). We also observed a TT insertion in the *mtrR* promoter, which shifts the −10/−35 spacing from the optimal 17 bp to 19 bp. This change in promoter spacing may exert a similar regulatory effect as the well-characterized single adenine deletion in the inverted repeat that reduces spacing to 16 bp, thereby increasing *mtrCDE* expression while repressing *mtrR* expression (42). Although these *mtrR* alterations are known to contribute to increased azithromycin MICs, they are not typically sufficient to account for the intermediate-level resistance observed (MIC = 16 µg/mL by Etest) (41). This level of resistance is more consistent with the presence of the 23S rRNA mutation C2611T (43) or 23S rRNA methylase genes such as *erm* (44), neither of which was detected in SLRRB23. Although the identified *pilQ* mutations, which reduce membrane permeability to antibiotics (45, 46), and the *mtrD* K823E substitution, which enhances efflux (41) (Table 3), may act synergistically with previously noted *mtr* locus mutations, the elevated azithromycin MIC could also reflect the contribution of additional, not-yet-characterized resistance mechanisms. We also explored the *porB* allele of SLRRB23, which did not match any existing NG-STAR classification, indicating it represents a novel variant. Notably, this new allele harbors the previously described A121D mutation, which has been associated with altered porin channel function (47). This mutation may reduce membrane permeability to hydrophilic antibiotics, including tetracycline and macrolides such as azithromycin, thereby contributing to the elevated MIC observed in SLRRB23 (47, 48). In combination with the *mtr* locus mutations and other efflux- and permeability-related changes, the *porB* A121D substitution could help explain the intermediate-level azithromycin resistance in the absence of 23S rRNA mutations or *erm* genes in this strain. Similarly, although SLRRB23 displayed high-level tetracycline resistance, we did not detect the *tetM* gene (49) or the chromosomal *rpsJ* V57M mutation (50), both of which are associated with MICs in the observed range. This again implies that other mechanisms could be contributing to tetracycline resistance in this strain.

**TABLE 3:** Genomic analysis of known AMR genetic determinants present in *Neisseria* sp. SLRRB23

Gene locus	Mutation(s)
*acnB*	K371Q
*gyrA*	S91T
*mtrC*	−35T→G
*mtrD*	K823E
*mtrR^a^*	D79N; mosaic
*penA*	A311V, A501T; allele type 59.001 (92.63% sequence identity)
*pilQ*	S341N, N648S
*porB*	A121D

^*a*^ Although not a previously characterized AMR determinant, the *mtrR* promoter spacer region contains a TT insertion that changes it from an optimal 17 to 19 bp.

## DISCUSSION

The identification of a commensal *Neisseria* species harboring a chromosomal copy of the DR-9 gonococcal marker underscores the complexities inherent in the molecular diagnosis of *Ng* infections. This finding represents only the second reported instance of such an occurrence, the first being documented in 2012 (7). While the first identified isolate was a *Neisseria macacae* strain that cross-reacted with the Roche cobas 4800 platform but not with the updated cobas 6800 platform (51), the newly identified *Neisseria* species cross-reacts with both of these cobas test systems. The presence of DR-9 in a non-gonococcal *Neisseria* species has significant implications for the specificity of gonococcal NAATs, particularly in cases where persistent positive results are obtained despite a negative culture for *Ng*.

Two primary hypotheses may explain the presence of the DR-9 marker in this commensal *Neisseria*. The first possibility is that this strain acquired the DR-9 marker from *Ng* by HGT through transformation (uptake of naked gonococcal DNA) or by infection and integration of the DR-9-containing prophages itself, either during coinfection in this patient or in a previously coinfected host. Since we could not test for the presence of gonorrhea DNA in the original NAAT specimen before antibiotic treatment, as it was discarded promptly after testing, it is possible that the patient had an initial low-level gonococcal infection. Commensal *Neisseria* species are naturally competent and can acquire genetic material from related species through transformation (52). The high degree of genetic similarity between *Ng* and commensal *Neisseria* facilitates such exchanges, making HGT a plausible mechanism for the acquisition of DR-9 (6). Additionally, gonococcal phages have been shown to infect a variety of gram-negative bacteria (53). Broadly, *Neisseria* prophages have been predicted to have a wide host range among commensal and pathogenic *Neisseria,* acting as mobile genetic elements, which could mediate HGT within this genus (54). The DR-9 region is repeated thrice within the genome of most gonococcal strains and lies within gonococcal filamentous phages NgoΦ1, 2, and 3 (55). In this regard, gonococcal NgoΦ1 has been shown to replicate and form phage particles that are released from the bacterial cells; although attempts to form plaques on *N. subflava, N. lactamica,* and *N. cinerea* with this phage could not be demonstrated (55). Given that the DR-9 region identified in *Neisseria* sp. SLRRB23 shares only 92% similarity with the gonococcal DR-9 sequence (Fig. 2), and that the patient tested negative for *Ng* at the urethral and rectal sites, if this commensal species acquired the gene from *Ng*, it likely occurred well before this patient and began to diverge over time. It is also possible that this commensal species directly acquired the DR-9 prophage region from another uncharacterized commensal species, which at some point in the past acquired it from *Ng*. A second possibility is that this previously undescribed *Neisseria sp*. evolved the DR-9 region from a shared common ancestor. Both the *Ng* and SLRRB23 DR-9 regions are located within *Caudoviricetes*-associated genomic regions, which are a class of tailed bacteriophages. However, given the relatively high sequence similarity between the SLRRB23 DR-9 and the gonococcal DR-9 sequences, along with the markedly different sequence similarity in the flanking regions surrounding the SLRRB23 DR-9 sequence (Fig. 2), it seems the most likely explanation is that the DR-9 element was acquired from *N. gonorrhoeae* through HGT. As it is, our ability to understand the origins of the DR-9 sequence in this commensal strain remains limited, given the lack of characterized commensal *Neisseria* genomes. While pathogenic *Neisseria* are well characterized, having over 45,000 published genomes for *N. gonorrhoeae* and over 16,000 for *N. meningitidis* in the NCBI Genome database, there are only 1,592 published nonpathogenic *Neisseria* genomes (as of 19 May 2025), making our ability to truly assess analytic specificity inadequate.

The oropharynx, a common site for commensal *Neisseria* colonization, presents a unique challenge for diagnostic assays. The genetic diversity of commensal *Neisseria* in this niche increases the risk of cross-reactivity in gonococcal NAATs (8). In addition, the role of commensal *Neisseria* species as reservoirs of AMR genes further complicates the landscape. These species can harbor resistance determinants that, through HGT, may be transferred to pathogenic *Neisseria*, contributing to the emergence of resistant strains (6, 56, 57). Specifically, this newly discovered commensal strain showed a concerning AST profile and an array of known and new genomic marker determinants of AMR. Surveillance of AMR profiles in commensal *Neisseria* is therefore critical for early detection and mitigation of resistance spread (58).

Despite the high analytic specificity of assays like the Roche cobas CT/NG NAATs (9, 36, 51, 59), the potential for cross-reactivity with commensal *Neisseria* species remains a possibility and should be considered in cases when a false-positive is suspected. Persistent positive results, particularly from oropharyngeal samples, are encouraged to undergo secondary testing to confirm *Ng* infection (11). Some countries and jurisdictions have advocated for confirmatory testing, whereby extragenital samples testing positive in a screening CT/NG NAAT are confirmed by a second NAAT assay that targets a different genetic marker or uses a different set of primers (60–62). While some commercially available NAATs do not specify the gene target, several would be appropriate reflex tests in cases of a suspected false-positive on the Roche cobas CT/NG NAATs; these include Hologic Aptima Combo 2, which targets the 16s rRNA gene, Abbott RealTime CT/NG test, which targets the *opa* genes, and Becton Dickinson ProbeTec GC Qx, which targets the *Ng piv* genes encoding pilin gene inverting protein homologs (63–65). However, having two distinct testing platforms for these cases is costly and, in most cases, not feasible.

To combat these barriers to implementation, utilizing NAATs that integrate dual targets within a single primary diagnostic assay offers significant advantages for extragenital testing. Dual-target assays, which produce a positive result if either target is present, can improve sensitivity, especially in cases of diagnostic escape (66). Comparatively, dual-target assays, which only produce a positive result if both targets are present, can improve specificity and reduce the likelihood of cross-reactivity with commensal *Neisseria* species (66–68). The dual-target approach can streamline the diagnostic process, minimize patient recall for additional testing, and expedite appropriate treatment initiation (68, 69). Such strategies are particularly beneficial in settings where follow-up testing may be logistically challenging or where patient compliance is a concern (69). While the cobas CT/NG targets two conserved regions in the *Ng* DR-9, the assay does not discriminate between them, and the second target is a variant DR-9 sequence that is not present in most *Ng* strains (36, 59, 70). In the USA, only two commercially available NAATs (Cepheid Xpert and Becton Dickinson CT/GC/TV2), approved for the diagnosis of *Ng* in pharyngeal specimens, require two targets to be present for a positive result (71, 72). However, neither discloses the specific genes that are targeted, greatly restricting our ability to assess the suitability of these assays for our study. Additionally, there is no indication that either test flags single-target positives, which is crucial to surveil for diagnostic escape variants in *Ng*.

Alternatively, several laboratory-developed tests (LDTs) have been developed for in-house detection of *Ng* and can be adapted to run on certain commercial NAAT platforms (11). Many of these tests target the *porA* pseudogene, first identified in 1998 (73), which is a highly conserved pseudogene homologous to the *porA* gene found in *Neisseria meningitidis* (74). Its nearly universal presence in all *Ng* strains tested makes it a highly valuable diagnostic target (75). However, *Ng* strains containing recombinant *porA* genes and producing false-negative results on in-house *porA* LDTs have been described worldwide (76–79) and have recently reemerged (80). Other common diagnostic gene targets include *opa*, 16S rRNA, *piv*, and *cppB* (11, 63, 64, 81), but it is important to note that each of these targets has limitations. Given that there is no perfect diagnostic target, implementation of dual-target and/or confirmatory assays can serve as a tool in the rare, albeit inevitable, case of a diagnostic escape variant or cross-reactive nongonococcal strain.

There are several limitations of this study to note. First, as mentioned above, we were unable to test the original positive NAAT from the patient before initial antibiotic treatment because it was discarded as per the testing laboratory’s policy. This reduces our ability to confidently say that the patient was never infected with *Ng*. Second, we only tested this isolate on the Roche cobas and Aptima Combo 2 platforms. Although Roche cobas is the only NAAT that targets DR-9, we cannot be certain that this strain does not cross-react on any other tests besides the AC2 test, which provided a negative result. We chose the AC2 platform as an alternate target because it was readily available to us at the CDC. Third, we did not extensively characterize this strain’s biochemical, morphological, and microbiological properties. Future work is needed to better understand the complete taxonomic characterization of this commensal *Neisseria* strain and its role in the human oropharynx.

In conclusion, the detection of the gonococcal DR-9 marker in a commensal *Neisseria* species highlights the need for ongoing vigilance in the molecular diagnosis of *Ng*. Understanding the mechanisms of genetic exchange and the role of commensal species in AMR gene dissemination is vital for developing accurate diagnostic tools and effective treatment strategies.

## Data Availability

The genome and annotation files are available in GenBank under accession number NZ_JBPBFH000000000.

## SUPPLEMENTAL MATERIAL

File S1 jcm.01022-25-s0001.fasta FASTA file containing the *de novo* genome assembly of *Neisseria* sp. SLRRB23.

Supplemental figures and tables jcm.01022-25-s0002.docx Figure S1 and Table S1 to S3.

Table S2 jcm.01022-25-s0003.xlsx BLAST search in the PubMLST database of the *N. meningitidis ggt* gene sequence among reported non-pathogenic *Neisseria* spp. Genomes (using the Not *Neisseria meningitidis* AND Not *Neisseria gonorrhoeae* syntax).

Table S3 jcm.01022-25-s0004.xlsx Average nucleotide identity (ANI) among human commensal and pathogenic *Neisseria* species using fastANI and pyANI methods.

ASM does not own the copyrights to Supplemental Material that may be linked to, or accessed through, an article. The authors have granted ASM a non-exclusive, world-wide license to publish the Supplemental Material files. Please contact the corresponding author directly for reuse.
