# Supplementary material for: A novel commensal Neisseria species harboring the gonococcal diagnostic marker DR-9 causes false-positive Roche cobas NAAT results
Source: J Clin Microbiol. 2025 Nov 19;63(12):e01022-25. doi: 10.1128/jcm.01022-25 (PMC12710327; doi:10.1128/jcm.01022-25)
Supplement: Supplemental figures and tables — Figure S1 and Table S1 to S3. [file jcm.01022-25-s0002.docx]

**Supplemental Material**


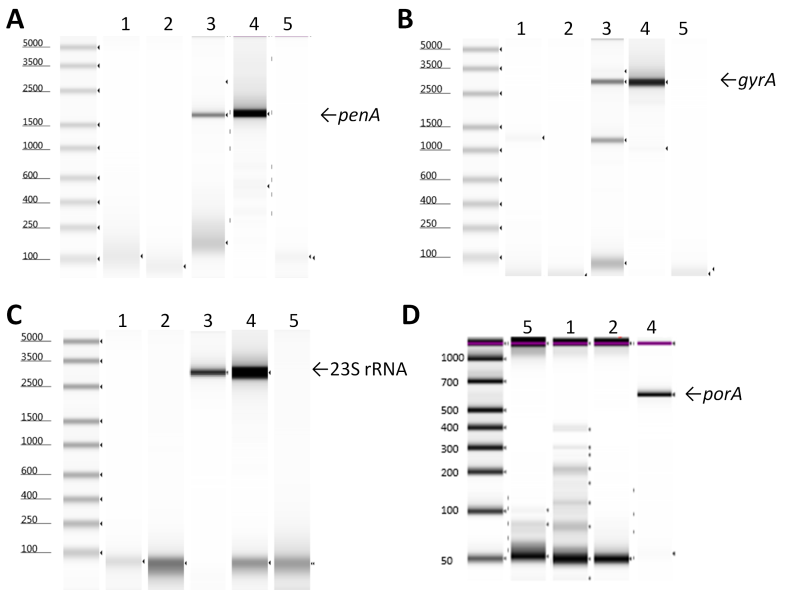


**Figure S1. Visualization of the PCR amplified gonococcal genes *penA* (A), *gyrA* (B), 23S rRNA (C) and pseudogene *porA* (D) from a remnant pharyngeal NAAT specimen from the patient.** The remnant pharyngeal NAAT (Lane 1) from the patient failed to amplify target bands for any of the gonococcal genes analyzed. Additional extracted DNA samples shown above are the following: (Lane 2) Extraction negative control (reagent blank), (Lane 3) Aptima Combo 2- and culture-confirmed gonorrhea-positive urine specimens for panel A (BioSample accession: SAMN29252141) and panel C (BioSample accession: SAMN29633225), and a pharyngeal swab specimen for panel B (BioSample accession: SAMN21923693), (Lane 4) genomic DNA of *Neisseria gonorrhoeae* isolates NCIP-12 for panels A, B, and C (BioSample accession: SAMN11309803) and GCWGS_4213 for panel D (BioSample accession: SAMN10425052), (Lane 5) Negative Template Control.

**Supplemental File 1.** FASTA file containing the de novo genome assembly of *Neisseria* sp. SLRRB23.

**Supplemental Table 1**. Primer sequences used to PCR amplify gonococcal gene regions.

| **Gene Target** | **Forward Primer Sequence** | **Reverse Primer Sequence** |
| --- | --- | --- |
| 23S rRNA | GGGATTTCAAGTTTGCTTACTTAGTCA | GCCTTAATCAATATATTCGGTAATGACTG |
| *gyrA* | GGCATAACCGCCGCACCGTTTC | GCTATATTGTGAGAAGCTGGATGAC |
| *penA* | CTGCATCAGGATAATAATAACGAGAAGT | GCCAAAGGGCTTAACTTGCTGAACAT |
| *porA* | TCAAGCCGCCTTCCCCATAGCCG | CAGCTCGAGCAAGACGTATCCGTTG |

**Supplemental Table 2.** BLAST search in the PubMLST database of the *N. meningitidis ggt* gene sequence among reported non-pathogenic *Neisseria* spp. Genomes (using the Not Neisseria meningitidis AND Not Neisseria gonorrhoeae syntax).

**Supplemental Table 3.** Average Nucleotide Identity (ANI) among human commensal and pathogenic *Neisseria* species using fastANI and pyANI methods.
